# Radiographic Imaging to Evaluate Food Passage Rate in Preterm Piglets as a Model for Preterm Infants

**DOI:** 10.3389/fped.2020.624915

**Published:** 2021-01-15

**Authors:** Susanne Soendergaard Kappel, Per Torp Sangild, Thomas Scheike, Christel Renée Friborg, Magdalena Gormsen, Lise Aunsholt

**Affiliations:** ^1^Comparative Pediatrics and Nutrition, University of Copenhagen, Copenhagen, Denmark; ^2^Department of Neonatology, Copenhagen University Hospital Rigshospitalet, Copenhagen, Denmark; ^3^Department of Pediatrics, Odense University Hospital, Odense, Denmark; ^4^Section of Biostatistics, Department of Public Health, University of Copenhagen, Copenhagen, Denmark; ^5^Department of Radiology, Copenhagen University Hospital Rigshospitalet, Copenhagen, Denmark

**Keywords:** enteral nutrition, feeding intolerance, gut motility, bovine colostrum, x-ray

## Abstract

**Objectives and study:** Gut motility in infants mature with increasing post-menstrual age and is affected by numerous hormonal, immunological and nutritional factors. However, it remains unclear how age and diet influence gut motility and its relation to feeding intolerance and gastric residuals in preterm neonates. Using preterm piglets as a model for infants, we investigated if contrast passage rate, as determined by X-ray contrast imaging, is affected by gestational age at birth, advancing postnatal age and different milk diets.

**Methods:** Contrast passage rate was evaluated using serial abdominal X-ray imaging on postnatal day 4 and 18 in preterm and near-term piglets fed infant formula, colostrum or intact bovine milk, with or without added fortifier (total *n* = 140).

**Results:** Preterm piglets had a faster small intestinal passage rate of contrast solution at day 4 of life than near-term piglets (SIEmpty, hazard ratio (HR): 0.52, 95%CI [0.15, 0.88], *p* < 0.01). Formula fed piglets at day 4 had a faster passage rate of contrast to caecum (ToCecum, HR: 0.61, 95%CI [0.25,0.96], *p* = 0.03), and through the colon region (CaecumToRectum, *p* < 0.05, day 4) than colostrum fed preterm piglets. The time for contrast to leave the stomach, and passage through the colon in day 4 preterm piglets were slower than in older piglets at day 18 (both, *p* < 0.05). Adding a nutrient fortifier increased body growth, gastric residuals, intestinal length and weight, but did not affect any of the observed passage rates of the contrast solution.

**Conclusion:** Serial X-ray contrast imaging is a feasible method to assess food passage rate in preterm piglets. Contrast passage rate through different gut segments is affected by gestational age at birth, postnatal age, and milk diet. The preterm piglet could be a good model to investigate clinical and dietary factors that support maturation of gut motility and thereby feeding tolerance and gut health in preterm infants.

## Introduction

Gut motility secures the continuous movement of nutrients through the gastrointestinal tract (GIT) via coordinated contractions of the smooth muscle layers, regulated by both dietary, neural, and hormonal factors (1). In newborn infants, the maturation stage of gut motility depends on post-menstrual age at birth. At 34–36 weeks gestation, the motility pattern reaches a relatively mature level, although some maturation of motility may continue throughout infancy and childhood in both term and preterm infants (1–3). Adequate physiological maturation of intestinal motility is important for preterm infants to tolerate enteral feeding and to avoid serious gastrointestinal (GI) complications, such as necrotizing enterocolitis (NEC). Early enteral feeding stimulates GI motor activity as shown by manometry studies of the immature intestine in preterm infants (4). This may occur via both physical food stimulation and luminal nutrients affecting the release of motility-promoting hormones, such as neurotensin from enteroendocrine cells in the small intestine (5).

Following preterm birth, mother's own milk or donor human milk is not always available and alternative enteral diets may be required. Diet type and nutrient density (e.g., breast milk, formula, human donor milk, nutrient-fortified human milk) vary widely for preterm infants and all these factors may influence food passage rate and maturation of gut motility patterns. In preterm piglets, bovine colostrum improves GI function compared with formula and human donor milk, as shown by reduced NEC incidence and improved digestive function and immunity (6–8). In ongoing clinical studies, the effects of bovine colostrum on growth, feeding intolerance, and defecation patterns (Amsterdam Stool Scale) are being investigated (9, 10) (Clinical trials: NCT03085277, NCT03537365).

To assess intestinal motility in preterm infants, previous studies have used food colors, abdominal ultrasound, manometry, or scintigraphy (11–14). Most of these studies can only assess motility of the upper or lower part of the GIT, without integrated information about the motility along the entire GIT. All these methods are technically difficult to perform because of the size and fragile condition of preterm infants, and some of them are relatively invasive and may interfere with the infant physiology (1). In clinical practice, standardized abdominal X-ray imaging is performed to evaluate functions in pathological conditions (e.g., obstruction, ileus, NEC), with or without the use of contrast solutions (15). Contrast solution provides detailed information about the gut transit time across different gut regions. This method, has not yet been used to provide information about the age- and diet-related maturation of gut motility in infants, partly because of the potential health risks associated with serial X-ray examinations.

Preterm piglets delivered at 90% gestation have become a well-established animal model of preterm infants to investigate gut development, nutrition, NEC and clinical complications of prematurity (16). Proof-of-concept studies in preterm pigs may pave the way for focused investigations in preterm infants and provide initial information about the factors that affect development of intestinal motility and food passage rate in infants. Using this model, we hypothesized that gestational age at birth, type of nutrition and age after birth are factors that influence contrast passage rate as assessed by X-ray imaging. To assess the possible utility of this method to monitor intestinal motility in immature newborns, we compared recordings from preterm and near-term cesarean-delivered piglets, from preterm piglets fed bovine colostrum or formula, and from piglets at different ages (4 and 18 days), with and without bovine colostrum used as a nutrient fortifier to cow's milk.

## Materials and Methods

### Animals

Preterm and near-term piglets were delivered from sows by cesarean section at 90% gestation (day 105–106 of gestation, 11 litters) and 96% gestation (113 days of gestation, 2 litters), which were used in 5- and 19-day experiments to investigate the effects of different enteral diets on gut structure and function (10, 17, 18). Piglets were placed individually in preheated incubators (37–38°Celcius) and received supplementary oxygen during the first 24 h (1–2 L/min). Two hours after delivery, a feeding tube was placed for enteral nutrition (EN) and a vascular catheter inserted via the umbilical cord for parenteral nutrition (PN). Piglets were orally fed every 2–3 h with gradually increasing volumes of either infant formula (*n* = 60) or dilute bovine milk (*n* = 50). The bovine milk fed was fed with or without fortification, using bovine colostrum powder, to increase protein and energy contents (e.g., protein 27–55 g/L, energy 2426-3602 kJ/L) (10). To ensure adequate fluid and nutrient intake, enteral nutrition was supplemented with PN to reach a total fluid intake of 120–180 ml/kg/d (Kabiven, Fresenius Kabi, Bad Homburg, Germany). In the 19-day study piglets were at day 7 transitioned to voluntary drinking from troughs. To secure that all piglets were healthy, they were continuously monitored by caretakers for clinical symptoms of feeding intolerance, hemorrhagic diarrhea, pain, and/or respiratory distress throughout the study periods. Pigs were euthanized, if they developed severe clinical symptoms. All animal studies were approved by the National Committee on Animal Experimentation, Denmark (license nr: 2014-15-0201-55 00418), and in line with the ARRIVE guidelines (19).

At time of organ harvest, one last bolus meal of 15 mL/kg was administered 60–90 min before tissue collection, when piglets were anesthetized and euthanised with an overdose of sodium pentobarbital. The gastrointestinal organs were removed and separated into stomach, small intestine and caecum-colon. The mass of the gastric residual was determined by weighing the stomach before and after emptying its contents. The small intestine was dissected by cutting along the mesenteric border and the intestine and colon wet weights were determined.

For Study 1, piglets were born preterm (*n* = 30) or close to term (*n* = 27) and fed increasing amounts of formula for 5 days (Table 1). For Study 2, preterm piglets were fed increasing amounts of bovine colostrum (*n* = 17) or formula (*n* = 33, Table 1), as described previously (17, 18). In study 1 and 2, radiographic recording was performed on day 4. Finally, for Study 3, preterm piglets (*n* = 33) had radiographic recordings done taken both on day 4 and 18 (Table 1). Some of these piglets were fed fresh dilute cow's milk (control, *n* = 17) while others were fed the same milk fortified with bovine colostrum powder (*n* = 16), as described previously (10). As the present study aimed to investigate effects of gestational age, maturity at birth and milk diets on intestinal passage rate under normo-physiological, healthy conditions, all piglets with signs of NEC or other severe complications were excluded prior to X-ray examination. We have previously reported the effects of NEC development on gastric residuals and contrast passage rates, as assessed by X-ray imaging (20).

**Table 1:** Clinical outcomes.

	**Experiment 1**			**Experiment 2**			**Experiment 3**		
	**Preterm**	**Near-term**	***P***	**Colostrum**	**Formula**	***P***	**Fortified**	**Non-fortified**	***P***
Total *n*	30	27		17	33		16	17	
Birth weight (g)	1,028 ± 219	1,078 ± 262	NS	1,084 ± 198	1,007 ± 229	NS	1,024 ± 161	1,009 ± 214	NS
End weight (g)	1,114 ± 195	1,179 ± 309	NS	1,224 ± 249	1,095 ± 209	NS	1,954 ± 331	1,502 ± 279	<0.01
Passage of meconium (h)	31 ± 18	37 ± 27	NS	30 ± 19	31 ± 17	NS	25 ± 11	27 ± 16	NS
Gastric residual (g/kg)	16 ±9	19 ± 12	NS	19 ± 6	17 ± 9	NS	12 ± 2	8 ± 3	<0.01
Intestinal length (cm)	483 ± 153	471 ± 91	NS	327 ± 47	466 ± 150	<0.01	473 ± 51	405 ± 41	<0.01
Intestinal weight (g)	35 ± 6	37 ± 10	NS	37 ± 8	37 ± 15	NS	75 ± 12	54 ± 14	<0.01
Relative intestinal weight (g/kg)	32 ± 4	31 ± 4	NS	31 ± 5	34 ± 16	NS	39 ± 4	37 ± 11	NS
Colon weight (g)	11 ± 3	15 ± 3	<0.05	14 ± 5	11 ± 3	NS	33 ± 15	25 ± 7	NS
Relative colon weight (g/kg)	10 ± 1	11 ± 1	NS	11 ± 4	11 ± 3	NS	17 ± 7	18 ± 7	NS

### Contrast X-Ray Imaging

**Figure 1:**
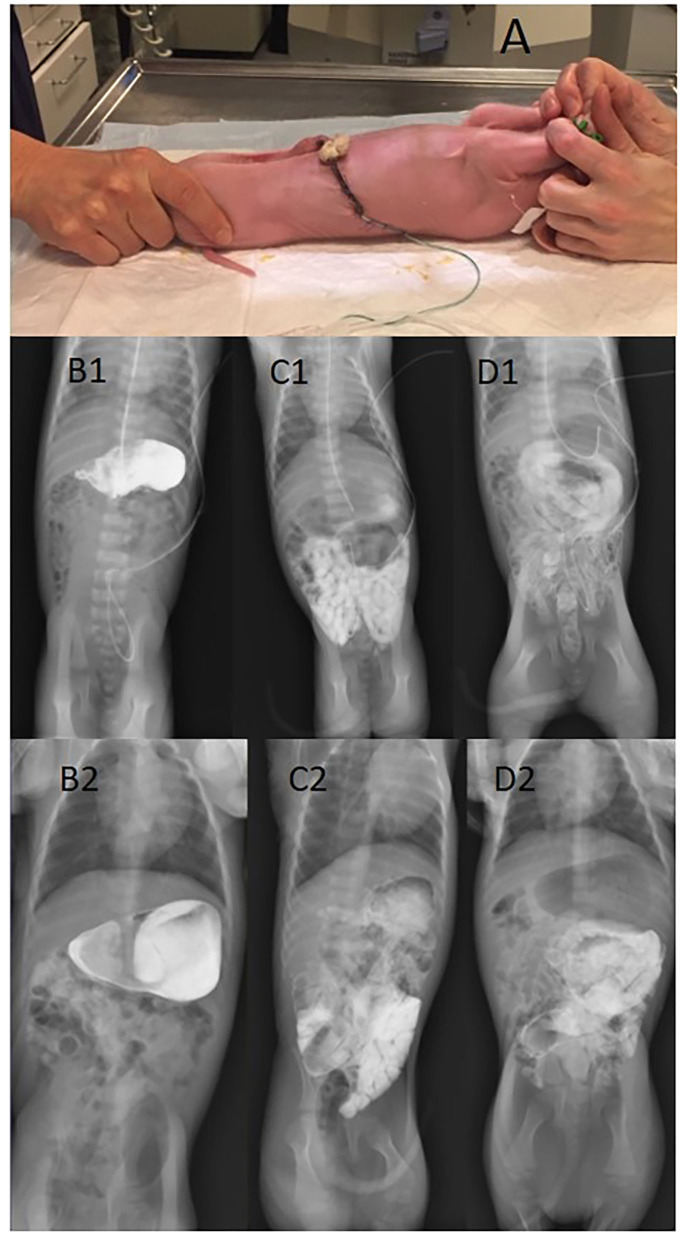
Contrast X-ray imaging of preterm piglets placed in lateral recumbence for taking the X-ray picture **(A)**. The figure also shows a stomach filled with contrast solution **(B1, B2)**, contrast reaching the caecum **(C1, C2)**, and contrast in colon and rectum with a near-empty small intestine **(D1, D2)**. Upper panel: 4 days of age; lower panel: 18 days of age.

Luminal passage of contrast through the GI tract was assessed by transit time recordings for the contrast solution on day 4 or day 18. Briefly, piglets were fasted for 2–3 h and then administered 4 mL/kg contrast (Iodixanol, Visipaque, GE Healthcare, Brøndby Denmark) by a feeding tube. Piglets receiving contrast on day 18, ate the contrast solution from a trough, to minimize the risk of lung aspiration and the potential effect on gastric motility by insertion of the feeding tube. Abdominal X-ray images were captured (80 kV/1,00 mAs) using Mobilett XP Hybrid (Siemens, Germany) at 10, 25, 60, 120, 180, 240 min after contrast was given and every 120 min until the contrast had left the gut completely. The piglets were fed with their normal enteral diet (colostrum, formula or cow's milk) 25 min after intake of the contrast solution and fed every 3 h during X-ray examination. X-rays were performed with piglets restricted and placed in supine position, see Figure 1. The procedure lasted ½-1 min and X-ray images were stored digitally. Contrast passage rate was evaluated by a neonatologist and an expert radiologist blinded to age and diets of the piglets. The time periods taken for the contrast to leave the stomach (StEmpty), reaching the cecum (ToCecum), emptied from small intestine (SiEmpty), and reaching the rectum (ToRectum) were recorded. Time taken for the contrast solution to pass through the colon (CecumToRectum) was calculated as the difference in time between ToCecum and ToRectum times.

### Statistics

Survival analyses for interval-censored data was used to analyse time-to-event outcomes. When analyzing time-to-event data, the time of interest lies in an interval. In this study we only know whether the time of interest occurred or did not occur at the time of X-ray imaging. If the time of interest did not occur at the latest X-ray imaging, then this is a right censored observation (21).

Comparing preterm with near-term piglets (Study 1), different diets (Study 2) and unfortified with fortified cow's milk (Study 3), interval-censored data was analyzed by the Cox regression model using the R-package icenReg (Statistical software R version 2.0.14). In addition, we validated the results by extensive simulations and other methods for discrete interval-censored data, considering that the intervals were based on observing the piglets at particular time points, as previously described (22). Results are presented as hazard ratio (HR) with 95% confidence interval. To compare StEmpty, ToCecum, CecumToRectum, SiEmpty, and ToRectum at day 4 and 18 (Study 3), we used the paired McNemar test. This was feasible because we had equivalent evaluation times for each piglet. Demographic/clinical data and time for first passage of meconium were analyzed using *t*-test and a non-parametric Wilcoxon test. We did not perform treatment-related power calculations for this initial method validation study because variation of the key outcome variables was unknown. Sample size was based on those sufficient to show age and diet effects in our previous studies (16). A *p* < 0.05 was considered statistically significant different and a *p* < 0.1 was considered as a tendency toward an effect (23).

## Results

Comparisons between groups in Experiments 1–3 were based on a total of 140 recordings (Table 1) from a total of 110 piglets. All piglets tolerated the X-ray examination well, with no obvious pain or distress during or after the 1 min handling for X-ray (Figure 1). The fecal score before intake of contrast was lower than after intake of contrast, indicating some laxative effect of contrast and/or handling (*p* < 0.01).

### Study 1: Preterm vs. Near-Term Piglets

**Figure 2:**
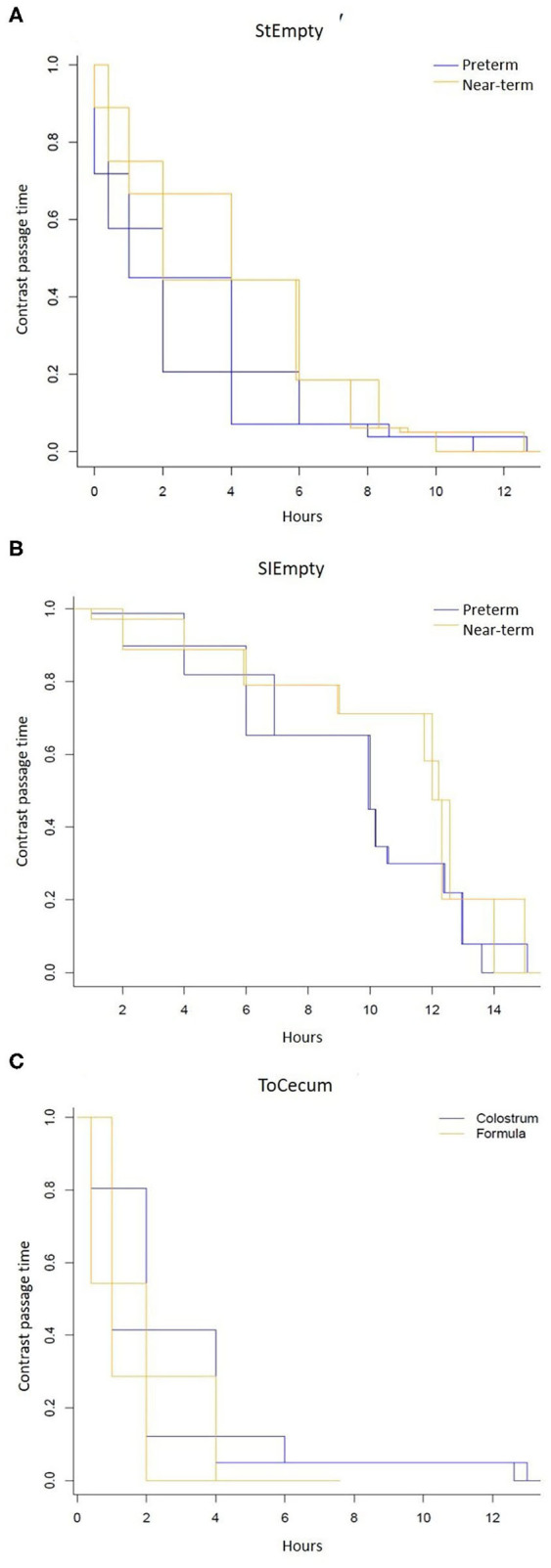
Contrast passage rate measured by serial of x-ray images after intake of contrast solution in 4 d-old immature piglets. **(A)** Stomach emptying time (StEmpty) in preterm and near-term piglets. **(B)** Small intestinal emptying time (SIEmpty) in preterm and near-term piglets. **(C)** Time for the contrast to reach the caecum (ToCaecum) in piglets fed formula or bovine colostrum.

Preterm and near-term piglets showed similar organ weights and length, time for first meconium passage and gastric residuals (Table 1), while colon weight tended to be higher in near-term piglets. For preterm piglets there was a tendency for contrast to leave the stomach at a slower rate compared with near-term piglets (StEmpty, HR: 0.67, 95%CI [0.24, 1.09], *p* = 0.1, Figure 2A). Time for contrast to leave the small intestine was faster in preterm piglets than in near-term piglets (SIEmpty, HR:0.52, 95%CI [0.15,0.88], *p* < 0.01, Figure 2B), while there were no differences in the time to reach caecum (ToCaecum, HR: 1.32, 95%CI [0.52, 2.11], *p* = 0.4) or rectum (ToRectum, HR:1.34, 95%CI [0.34,2.41], *p* = 0.5). The contrast passage rate through the colon (CaecumToRectum) was slower in preterm vs. near-term piglets (*p* < 0.05).

### Study 2: Bovine Colostrum vs. Formula for Preterm Piglets

There were limited differences between the two diets with regards meconium passage, residuals and intestinal weights on day 5 (Table 1). The intestinal length was greater in formula-fed piglets. On day 4, similar values across the diets were found for stomach emptying time (StEmpty, HR:0.97, 95%CI [0.41, 1.52]), *p* = 0.9), emptied small intestine (SiEmpty, HR:0.64, 95%CI [0.08, 1.20], *p* = 0.2) and time to reach to rectum (ToRectum, HR:0.85, 95%CI [0.135, 1.56], *p* = 0.7). Contrast passage rate was faster in the formula-fed piglets compared with colostrum-fed piglets (ToCecum, HR:0.61, 95%CI [0.25, 0.96], *p* = 0.03, Figure 2C). Likewise, the contrast passage rate through the colon region (CaecumToRectum) was faster in formula-fed vs. colostrum-fed piglets (*p* < 0.05, paired McNemar test).

### Study 3: Preterm Piglets Tested on Day 4 and 18, With and Without Milk Fortification

Nutrient fortification increased the growth of preterm piglets, also inducing higher relative weights and lengths of the small intestine, together with larger gastric residuals on day 19 (Table 1). On day 4, time for contrast to leave the stomach was slower compared with day 18 (*p* < 0.05). There were no differences in time for the contrast to reach cecum or rectum between day 4 and 18 (ToCeacum, *p* = 0.3 and ToRectum, *p* = 0.2). However, the calculated colon transit time was slower in day 4 piglets compared with day 18 (*p* < 0.05, CaecumToRectum) when assessing the time to events. For comparison between the dilute and the fortified cow's milk, the stomach emptying time (StEmpty, day 4: HR:0.83, 95%CI [0.13, 1.78], *p* = 0.7; day 18: HR: 0.49, 95%CI [0, 1.60], *p* = 0.4), intestinal transit time (Tocecum, day 4: HR:0.67, 95%CI [0.07, 1.42], *p* = 0.4; day 18: HR:3.58, 95%CI [0–25.5], *p* = 0.8), and time to reach to rectum (ToRectum, day4: HR:2.16, 95%CI [0.05, 4.37], *p* = 0.3; day 18: HR: 1.79, 95%CI [0.7,4.3], *p* = 0.5) were similar between the two groups on day 4 and day 18.

## Discussion

Feeding intolerance (FI) and poor gastrointestinal motility is a challenge in the clinical care of preterm infants. Adverse clinical conditions, such as sepsis or gut inflammatory reactions, potentially leading to NEC, affect intestinal motility patterns, as indicated in our previous study (20). On the other hand, poor gut motility, and impaired food passage may be an inherent sign of physiological immaturity at birth, independent of clinical conditions and diseases, predisposing to large gastric residuals, NEC and sepsis. These interacting factors are difficult to study in infants. Using preterm piglets as a model, we now demonstrate that contrast passage rate is influenced by gestational age at birth, type of milk diet and postnatal age. We conclude that contrast X-ray imaging is a feasible method to investigate food transit rate in piglets, demonstrating region-dependent effects of gestational age at birth, postnatal age and milk diets. This adds information to the few studies in preterm infants on this topic, using a range of other methods (11–14).

Preterm piglets tended to empty their stomach at a slower rate than piglets born close to term. This result concurs with some previous infant perfusion manometry studies (4), indicating maturational changes in proximal gut motility with increasing gestational age at birth (2), and the common clinical observation of feeding intolerance, with large pre-feeding gastric residuals, in the early life of very preterm infants. Likewise, the slower contrast passage rate through the colon in preterm vs. near-term pigs may reflect the frequently reported risk of constipation in preterm infants, reflecting poor hind gut motility (24). Conversely, the faster passage rate through the small intestine in preterm vs. near-term individuals may reflect an inability of the immature intestine to promote an adequately controlled intestinal motility pattern, allowing maximal nutrient absorption, thereby predisposing to nutrient maldigestion (25).

Stomach and colon transit times were longer in piglets shortly after birth, relative to later (day 4 vs. 18), indicating maturational changes, and possibly feeding effects, also in preterm pigs. The results are in accordance with a study in preterm infants, showing that volume, and content of enteral intake increase the frequency of stool passage, independent of the digestibility of the enteral diet (26). Using ultra-sonographic imaging, one preterm infant study showed that bovine-milk-based fortified mother's milk reduced stomach emptying time (27). We observed more gastric residuals in fortified piglets (bovine milk fortified with bovine colostrum) but no difference in stomach emptying time assessed by x-ray contrast imaging. This may reflect that both diet type and density, together with maturation of intestinal motility pattern, affect food passage rates. We cannot exclude that motility effects of bovine colostrum differ from those of standard fortifiers, and the nature of the base diet may also be important (e.g., bovine milk vs. human milk). An ongoing infant study using bovine-colostrum-based fortifier to human milk may help to answer some questions for preterm infants (28).

In the first days after preterm birth, previous studies show that bovine colostrum improves gut functions in pigs, relative to formula, as assessed by less gastric residuals, improved digestive enzyme activities, immunological functions and NEC resistance (7). In this study, we observed no diet-related differences in intestinal wet weight but a longer intestine in formula-fed pigs. This may reflect a longer and thinner intestine in piglets fed formula, consistent with previous observations of structural changes in the gut of formula- vs. colostrum-fed piglets (29). Further, we found no effect of colostrum on contrast passage rate through the gut across piglets without NEC lesions. Since we assessed only healthy preterm piglets, this might indicate that previous observations of altered motility in formula-fed animals that developed mild NEC symptoms, could be a direct result of NEC development and the associated pro-inflammatory effects, rather than a specific effect of diet composition (20, 30). An ongoing clinical trial in preterm infants will help to show if bovine colostrum or formula, used in the first feeds after preterm birth, when mother's own milk is not available, influence gastric residuals, bowel habits, constipation and time to full enteral feeding (ClinicalTrials.gov Identifier: NCT03085277).

In infants, carmine red has been used in several studies to investigate the total transit time through the GIT (12). However, this approach does not accurately reflect food transit, but rather transit of the marker compound used. Furthermore, the osmolarity of the compound and the composition of the nutrient intake (orally or via a feeding tube) may impact the results obtained. In our study, we investigated the total transit time, i.e., from the first intake of our non-absorbable contrast solution (Iodixanol) to its entry into the rectum. We found that intake of contrast affected the fecal score and increased the transit time, indicating that the contrast solution itself may affect the total transit time. The osmolarity of the solution used in this study was 290 mOsm/L (unpublished observations) and therefore comparable to mother's milk (277-303 mOsm/l (31). Its chemical carrier constituents include mainly calcium and sodium which should not have notable direct gut osmotic effects or increase the fecal score, although such effects cannot be excluded (32). Digestive effects of a contrast or color solution are avoided when using Tc^99m^-DTPA scintigraphy that have also been used for preterm infants (13), despite the ethical limitations in using radioactive constituents. Most scientific methodologies related to gut motility and food passage studies in preterm infants, from color markers to use of serial X-rays or radiolabeled components, have ethical constraints that limits their use in clinical practice.

We used preterm piglets as a model for preterm infants because they show many similarities to infants in their complications after preterm birth, including maldigestion, poor respiration, brain defects, high NEC sensitivity and a degree of feeding intolerance with large gastric residuals (16, 20, 33, 34). The model has translational limitations, not only because preterm piglet anatomy and physiology may differ from infants (e.g., a relatively long small intestine and high NEC sensitivity) but also because serial X-rays are required to accurately assess the passage of contrast through the entire gut. Regardless, preterm piglets may be used effectively to study in detail the influence of medical, nutritional and disease-related factors that affect enteral feeding tolerance and the important transition from parenteral to full enteral feeding in immature newborn infants. We conclude that gestational age at birth, postnatal age, and milk diet influence intestinal motility in piglets, as evaluated by contrast passage rate and X-ray images. The method used was feasible and provided novel information about regional intestinal motility but may not be applicable to human preterm infants. Regardless, there is a great need to investigate different aspects of intestinal motility and food passage rate in human preterm infants, considering the important clinical problem of both primary (immaturity) or secondary (response to complications) intestinal dysmotility in preterm infants.

## Data Availability Statement

The raw data supporting the conclusions of this article will be made available by the authors, without undue reservation.

## Ethics Statement

The animal study was reviewed and approved by National Committee on Animal Experimentation, Denmark.

## Author Contributions

SK, PS, and LA: conception and design and data interpretation. SK, CF, MG, and LA: data acquisition. TS: data analysis. SK: writing original draft. TS, PS, and LA: critical review and editing. All authors are approval of the final manuscript.

## Conflict of Interest

The authors declare that the research was conducted in the absence of any commercial or financial relationships that could be construed as a potential conflict of interest.
